# Assessment of minimum clinically important difference in symptoms and functionality of Japanese patients with major depressive disorder following inadequate response to antidepressants: a *post hoc* analysis of the long-term study of brexpiprazole augmentation therapy in Japanese patients with major depressive disorder

**DOI:** 10.3389/fpsyt.2025.1556470

**Published:** 2025-03-26

**Authors:** Hikaru Hori, Masako Shiosakai, Yoshiyuki Shibasaki, Kentaro Yamato, Yilong Zhang

**Affiliations:** ^1^ Department of Psychiatry, Faculty of Medicine, Fukuoka University, Fukuoka, Japan; ^2^ Clinical Development, Headquarters of Clinical Development, Otsuka Pharmaceutical Co., Ltd., Tokyo, Japan; ^3^ Medical Affairs, Otsuka Pharmaceutical Co., Ltd., Tokyo, Japan; ^4^ Department of Public Health, Graduate School of Medicine, Juntendo University, Tokyo, Japan

**Keywords:** major depressive disorder, minimum clinically important difference, Montgomery-Åsberg depression rating scale, Sheehan disability scale, EuroQol 5-dimension 5-level

## Abstract

**Background and objectives:**

The aim of this study was to apply the minimum clinically important difference (MCID) concept to clinical results for Japanese patients with major depressive disorder following inadequate response to antidepressants, and to explore the disparity in what physicians and patients considered important in the treatment of depression.

**Methods:**

The original study was a 52-week, open-label, multicenter study on the administration of 2 mg/d of brexpiprazole as adjunctive therapy for patients with major depressive disorder. Here, we conducted a *post hoc* analysis to determine the MCID in Montgomery–Åsberg Depression Rating Scale (MADRS), Sheehan Disability Scale (SDS), and EQ-5D-5L-derived utility score. We compared the area under the curve (AUC) and correlation coefficients for the MADRS, SDS, and utility scores between the physicians’ and patients’ responses.

**Results:**

The MCIDs for this patient group were 4.89–4.94 for the MADRS score, 31.15–35.10% for the MADRS improvement rate, 0.69–2.14 for the SDS score, and 0.045–0.195 for the utility score. The MCIDs for the SDS and utility scores derived from the patient-perspective anchor were almost twice as high as those from the physician-perspective anchor. The utility score had the highest AUC and correlation coefficient for the patient-perspective anchor, while the MADRS score did for the physician-perspective anchor.

**Conclusions:**

The MCIDs for the MADRS, SDS, and EQ-5D-5L -derived utility scores were estimated. Physicians focus more on depressive symptoms and prioritize symptom severity over improvements in functionality and activities of daily life, in contrast to patients, who prioritize such improvements.

## 1 Introduction

Major depressive disorder (MDD) is a chronic, recurrent psychiatric disorder that causes significant morbidity and mortality (1). This disorder is associated with substantial social and economic costs in Japan, largely because of reduced workplace productivity and suicides (17). However, treatment responses are inadequate, with antidepressant monotherapy failing in approximately 50% of cases. Additionally, remission rates are lower than 40% with antidepressant monotherapy and efficacy tends to decline with subsequent lines of treatment (2). Lack of remission leads to ongoing disruptions in well-being, social functioning, quality of life, and broader social and economic impacts (21).

The Montgomery–Åsberg Depression Rating Scale (MADRS), Sheehan Disability Scale (SDS), and EuroQol 5-dimension 5-level (EQ-5D-5L) questionnaire are reliable tools for assessing symptoms, social functioning, and quality-of-life outcomes in MDD (3–5). MADRS, SDS and EQ-5D-5L are based on summary rating scores and lack a gold standard to interpret results. Physicians have to rely on the experience with individual patients and populations to interpret scores and the clinical significance of various degrees of change.

Most studies have focused on quantifying the efficacy of therapeutic interventions by reporting changes in group means before and after treatment. However, group means cannot be readily used in clinical practice to interpret changes in individuals, and statistical significance does not always translate to clinical significance, since large samples can lead to statistically significant differences that are not clinically meaningful (6). The concept of “minimum clinically important difference” (MCID) was therefore introduced as an alternative tool for quantifying clinically significant patient improvements due to therapeutic interventions. The MCID is defined as the smallest change that is meaningful to patients and is considered the threshold of treatment efficacy. If the MCID threshold is exceeded, the decision to treat is validated (7). Knowing the MCID is crucial for informing whether a treatment is effective in a way that is clinically meaningful to patients.

A 52-week, open-label, multicenter study previously demonstrated the favorable efficacy, safety, and tolerability profiles of 2 mg/day of brexpiprazole as an adjunctive therapy for patients with MDD (NCT01360866) [Kato et al., (18); Otsuka Pharmaceutical Co]. Here, we conducted a *post hoc* analysis of that study to determine the MCID values for the MADRS, SDS, and EQ-5D-5L-derived utility score. Since several studies have shown that physicians and patients differ significantly in what they consider important for “being cured” of depression, one of the aims of this study was to provide a more comprehensive assessment of MCID for patients with MDD following inadequate response to antidepressants, incorporating both physician and patient perspectives. Further, to understand whether there is alignment or divergence in the focus of disease improvement between physicians and patients, we evaluated the responsiveness of these outcome measures from both physician and patient perspectives.

## 2 Methods

### 2.1 Overview

This analysis was divided into two parts. First, we estimated the MCIDs for the MADRS, SDS, and EQ-5D-5L-derived utility score using data from an open-label clinical trial conducted in Japan, incorporating both physician and patient perspectives. Second, to understand whether there is alignment or divergence in the focus of disease improvement between physicians and patients, we analyzed the area under the curve (AUC) and correlation between MADRS, SDS, and utility scores between the patients’ and physicians’ perspective anchor.

### 2.2 Patient population

The primary study of this *post hoc* analysis was an open-label, multicenter, 52-week study conducted in Japan to evaluate the long-term safety and efficacy of adjunctive fixed-dose brexpiprazole 2 mg/day for the treatment of major depressive disorder (MDD) (NCT03737474). This *post hoc* analysis only enrolled rollover participants who had completed the 6-week, double-blind, randomized, placebo-controlled Phase 2/3 BLESS study (NCT03697603). Full details of both the BLESS study and the long-term study have been published previously.

Participants in this *post hoc* analysis were Japanese adults aged 20–64 years with a single or recurrent episode of MDD, diagnosed according to the Diagnostic and Statistical Manual of Mental Disorders, Fifth Edition (DSM-5) criteria, with a duration of ≥8 weeks. During the screening phase of the BLESS study, eligible patients were required to have received adequate treatment with 1–3 different antidepressants during their current MDD episode and to have had an inadequate response to each agent. Adequate treatment was defined as treatment with an antidepressant at an approved dose for ≥6 weeks. Inadequate response was defined as <50% improvement in patient self-evaluation of their previous antidepressant treatment, with 100% representing complete improvement and 0% representing no improvement in depressive symptoms.

Patients meeting these criteria and having a Hamilton Depression Rating Scale (HAM-D 17-item) total score of ≥18, indicating moderate to severe depression, were enrolled in a single-blind treatment phase. Following an 8-week treatment period, those with a HAM-D 17-item total score of ≥14 at Week 8 (indicating mild to severe depression) were randomized into the double-blind treatment phase. Patients who completed the Phase 2/3 BLESS study and opted to continue treatment were enrolled in the long-term study.

This *post hoc* analysis targeted these rollover participants and was conducted using the last observation carried forward (LOCF) dataset.

### 2.3 Outcome assessment

#### 2.3.1 MADRS

The MADRS is a widely used scale designed primarily to assess the psychiatric symptoms of depression and exclude somatic symptoms (20). It is widely recognized and used by US and European drug regulation agencies for measuring clinical efficacy in randomized controlled trials involving the treatment of MDD. It has been used as an outcome measure in numerous treatment efficacy studies and is increasingly adopted in clinical practice (3). Introduced by Montgomery and Åsberg in 1979, the MADRS consists of 10 items: apparent sadness, reported sadness, inner tension, reduced sleep, reduced appetite, concentration difficulties, lassitude, inability to feel, pessimistic thoughts, and suicidal thoughts. Each item is scored from 0 to 6, with the total score ranging from 0 to 60 (16). We calculated both the change in MADRS total score and the reduction in MADRS score after treatment.

#### 2.3.2 SDS

The SDS is a sensitive tool for identifying primary-care patients with mental health-related functional impairments who require a diagnostically oriented mental health assessment (4). This well-validated, brief, and simple self-report measure is easily administered in routine care and is used to assess the degree of functional impairment across three domains: work/school, family life/home responsibilities, and social/leisure activities (4). Patients rate the extent to which these items have been disrupted by their symptoms on a visual analog scale (VAS) from 0 (not at all) to 10 (extremely). Scores from the individual domains are combined to generate the SDS total score, which ranges from 0 (unimpaired) to 30 (highly impaired) (19).

#### 2.3.3 EQ-5D-5L

The EQ-5D-5L questionnaire is a standardized instrument for measuring health-related quality of life that was developed by the EuroQol Group, and it is commonly used for the clinical and economic appraisal of healthcare interventions (5).It is a generic, preference-based instrument that is used to describe and evaluate health. It has been widely tested and used in both general and patient populations, and it is available in over 130 languages. The EQ-5D-5L questionnaire used in this study consists of two parts: Part 1 involves self-assessment in five domains (mobility, self-care, usual activities, pain/discomfort, and anxiety/depression) on a scale of 1 to 5 (1 = no problems, 2 = slight problems, 3 = moderate problems, 4 = severe problems, 5 = extreme problems); and Part 2 involves self-rating of health on a VAS (0 = worst health imaginable, 100 = best health imaginable). We calculated the utility score, from 0 (dead) to 1 (perfect health), using a Japanese version of the EQ-5D-5L value set (5).

### 2.4 Anchors

#### 2.4.1 Patient-perspective anchor

We used the VAS, a widely used generic measure of self-reported health status, as the anchor from the patient’s perspective. It is commonly used as an anchor because it reflects participants’ assessments of their own health changes. The participants were asked to draw a horizontal line indicating their current health status to an appropriate point between 0 (worst imaginable health state) and 100 (best imaginable health state). A change in score from LOCF to baseline of 7–17 points was defined as a minimal improvement, and one of −7 to −17 points was defined as a minimal decline. Changes of ≥ 18 points and ≤ −18 points were defined as maximal improvements and declines, respectively. “No meaningful change” was defined as a change in score of −6 to 6 points (8).

#### 2.4.2 Physician-perspective anchor

We used the Clinical Global Impression–Improvement (CGI-I) scale for anchor-based MCID calculations from the physician’s perspective. This scale has been used extensively as an anchor in previous studies (9, 10). By comparing the baseline condition at the start of this study, Physicians assessed patient improvement on an 8-point scale: 0 (not assessed), 1 (very much improved), 2 (much improved), 3 (minimally improved), 4 (no change), 5 (minimally worse), 6 (much worse), 7 (very much worse).

### 2.5 Approaches used to calculate the MCID

There is currently no gold standard methodology for estimating the MCID. Most methods fall into two categories: distribution-based and anchor-based. We used both approaches in this study: (1) “change difference,” an anchor-based method whereby the MCID was calculated as the difference in mean changes in outcome from baseline to 52 weeks(LOCF) between participants responding with “about the same” and those responding with “a little better” from both patient and physician perspectives; (2) “minimum detectable change” (MDC), which is defined as the minimum amount of change capturing true clinical change rather than mere variability associated with repeated measurements. MDC method characterizes the MCID as the smallest quantifiable change that surpasses the threshold of measurement error with a specified level of confidence, typically set at the 95% confidence level. Consequently, the MCID value corresponds to the upper limit of the 95% confidence interval for the average change score observed in the cohort identified as non-responders. We used the MDC at the 95% confidence level (MDC95) in the current analysis. We determined it based on the standard error of measurement (SEM) and adjusted it for the 95% confidence interval (7). The outcome results at baseline and the 52 weeks (LOCF) were calculated in this *post-hoc* analysis.

### 2.6 Consistency of each outcome measurement with the two anchors

We used receiver operating characteristic (ROC) curve analysis, which has the advantage of synthesizing information on sensitivity and specificity, to evaluate the consistency of the outcome measurements (MADRS score, MADRS reduction rate, SDS score, and EQ-5D-5L-derived utility score) with the anchors. We evaluated the accuracy of the ROC curve based on the AUC: 0.90–1.00, excellent; 0.80–0.90, good; 0.60–0.80, fair; and 0.50–0.60, failure. We used Spearman’s correlation coefficient (r) to determine relationships between responses to the anchor. This measure ranges from −1 to +1: a value near 0 indicates no (linear) correlation and a value near ±1 indicates a very strong correlation. We used the following guideline on the strength of the linear relationship as indicated by the value of r: < 0.3, poor; 0.30–0.59, fair; 0.60–0.79, moderate; and 0.80–0.99, very strong (22).

### 2.7 Statistical analysis

We performed all statistical analyses using SAS version 9.4 (SAS Institute, Cary, NC, USA). Unless stated otherwise, hypothesis tests were two-sided with a 0.05 significance level; a P-value < 0.05 was considered significant.

## 3 Results

### 3.1 Patient group

A total of 216 patients from the primary study were included in this analysis. Baseline demographics and clinical characteristics are presented in **Table 1**. Mean (SD) age was 40.7 (10.3) years, and of the patients, 61.6% (133/216) were male. The outcomes measures of baseline and 52 weeks (LOCF) are presented in **Table 2**. At baseline, the study sample had a mean score of 19.85 on the MADRS, indicating moderate depressive symptom severity.

**Table 1:** Patient demographics and clinical characteristics.

Variable	mean or n (% or SD)
Demographic characteristics	
Age, years, mean (SD)	40.7 (10.3)
Sex, male, n (%)	133 (61.6)
Body weight, kg, mean (SD)	66.8 (15.1)
BMI, kg/m2, mean (SD)	23.9 (4.3)
Clinical characteristics	
Duration of current MDD episode, months, mean (SD)	12.0 (18.1)
Age at first onset of MDD, years, mean (SD)	35.1 (10.5)

SD, standard deviation; BMI body mass index.

**Table 2:** Outcomes (MADRS, SDS, utility score, and MADRS reduction rate) at baseline and 52 weeks after treatment.

Outcome score	Baseline, mean (SD)	52 weeks after treatment, mean (SD)
MADRS	19.85	14.98
	(8.78)	(9.82)
SDS	4.84	4.23
	(2.33)	(2.60)
EQ-5D-5L-derived utility score	0.776	0.785
	(0.162)	(0.183)
MADRS reduction rate	—	0.181
		(0.625)

EQ-5D-5L, EuroQol 5-dimension 5-level questionnaire; LOCF, last observation carried forward; MADRS, Montgomery–Åsberg Depression Rating Scale; SD, standard deviation; SDS, Sheehan Disability Scale.

### 3.2 MCID threshold values for outcome measures

The comparison of the anchor- and distribution-based approaches yielded a narrow range of MCID threshold values for the MADRS score (4.89–4.94) and MADRS improvement rate (31.15–35.1%). In contrast, the ranges of MCID threshold values for the SDS and utility scores were wider: 0.69–2.14 and 0.045–0.195, respectively. Notably, the MCID threshold derived from the patient-perspective anchor was almost twice that derived from the physician-perspective anchor for both the SDS score (patient perspective: 1.24; physician perspective: 0.69) and the utility score (patient perspective: 0.093; physician perspective: 0.045) (**Table 3**).

**Table 3:** MCID threshold values.

	MCID calculation method	Outcome measure			
		MADRS	SDS	Utility score	MADRS reduction rate
Distribution method	MDC95	4.93	2.14	0.195	0.351
Anchor method	Change difference (VAS anchor)	4.89	1.24	0.093	0.350
	Change difference (CGI anchor)	4.94	0.69	0.045	0.312

CGI, Clinical Global Impression(physician-perspective anchor); EQ-5D-5L, EuroQol 5-dimension 5-level questionnaire; MADRS, Montgomery–Åsberg Depression Rating Scale; MDC95, minimum detectable change at the 95% confidence level; SDS, Sheehan Disability Scale; VAS, visual analog scale(patient-perspective anchor).

### 3.3 Consistency of each outcome measurement with the two anchors

We used the ROC curve to compare the four outcome measures (Utility score, SDS score, MADRS score, MADRS reduction rate) assessed in this study to identify the most valid and responsive measure of therapeutic efficacy from both the patient and physician perspectives. We also calculated the association between the responses to the anchor and the change in outcome measures (**Table 4**).

**Table 4:** Consistency of each outcome measure with the two anchors.

Parameter	VAS		CGI	
	AUC	Spearman	AUC	Spearman
MADRS	0.6659	0.4532 (P < 0.0001)	0.8079	0.7660 (P < 0.0001)
MADRS reduction rate	0.6950	0.4811 (P < 0.0001)	0.8577	0.8138 (P < 0.0001)
Utility score	0.7414	0.6669 (P < 0.0001)	0.5793	0.4479 (P < 0.0001)
SDS	0.7001	0.4926 (P < 0.0001)	0.5873	0.3922 (P < 0.0001)

AUC, area under the curve; CGI, Clinical Global Impression (physician-perspective anchor); EQ-5D-5L, EuroQol 5-dimension 5-level questionnaire; MADRS, Montgomery–Åsberg Depression Rating Scale; SDS, Sheehan Disability Scale; VAS, visual analog scale (patient-perspective anchor).

#### 3.3.1 Patient-perspective anchor

**Figure 1:**
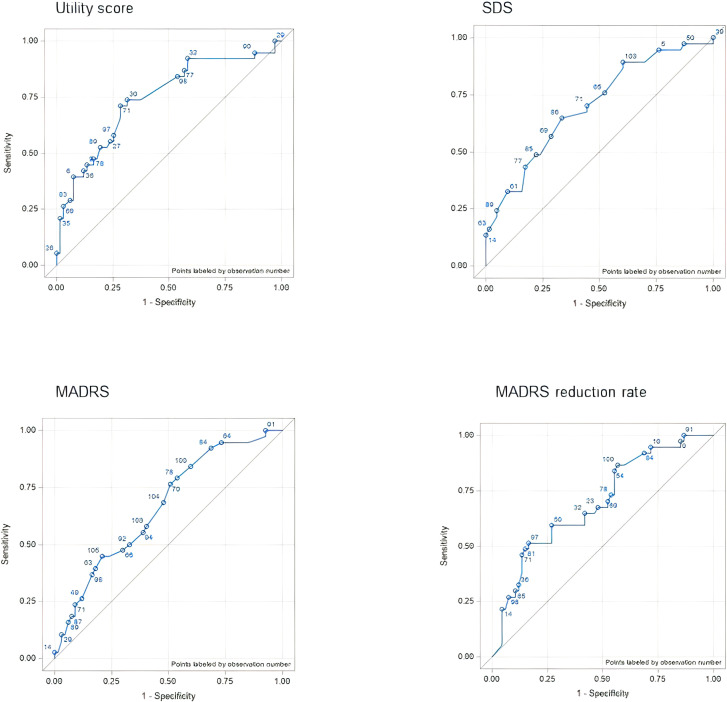
ROC curves for each outcome assessment according to the CGI anchor (physician perspective anchor). AUC, area under the curve; CGI, Clinical Global Impression; MADRS, Montgomery–Åsberg Depression Rating Scale; ROC, receiver operating characteristic; SDS, Sheehan Disability Scale.

The AUC varied from 0.6659 (change in MADRS score) to 0.7414 (change in utility score), indicating fair accuracy in discriminating between responders and non-responders. The correlations between the patient-perspective anchor and outcome measures were consistent with the AUC results. The strongest correlation was that between the response to the anchor and the utility score (r = 0.6669, P < 0.001), whereas the MADRS score was the most weakly correlated to the response to the anchor (r = 0.4532, P < 0.001) (**Figure 1**).

#### 3.3.2 Physician-perspective anchor

**Figure 2:**
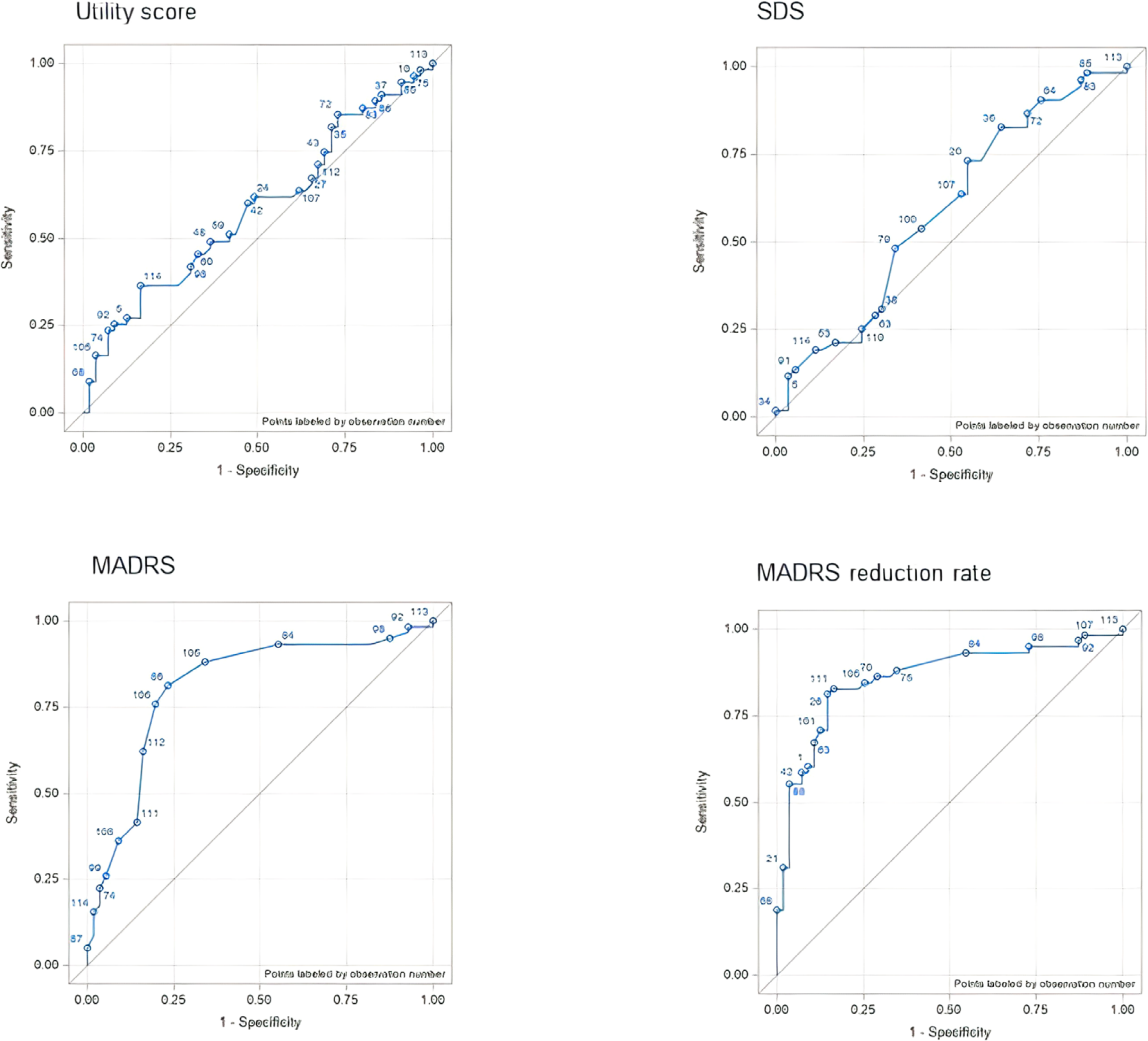
ROC curves for each outcome assessment according to the VAS anchor (patient perspective anchor). AUC, area under the curve; MADRS, Montgomery–Åsberg Depression Rating Scale; ROC, receiver operating characteristic; SDS, Sheehan Disability Scale; VAS, visual analog scale.

Compared to the patient-perspective anchor, there were larger differences in the AUC of the physician-perspective anchor among the outcome measures. The MADRS reduction rate had the highest AUC (0.8577) and the utility score had the lowest (0.5793). The Spearman correlation coefficients were consistent with the AUC results for the MADRS score (r = 0.7660, P < 0.001) and the MADRS reduction rate (r = 0.8138, P < 0.001). In contrast, the SDS score was most weakly correlated to the response to the anchor (r = 0.3922, P < 0.001) (**Figure 2**).

## 4 Discussion

The concept of the MCID was originally developed to interpret patient-reported outcomes, but it is gaining popularity in pharmacological treatment interventions. In this study, we aimed to estimate MCID threshold values for MADRS, SDS, and EQ-5D-5L-derived utility scores. Further, to explore the disparity between physician and patient perspectives, we compared the MCIDs and correlations between measures from both patient- and physician-perspective anchors.

### 4.1 MCID threshold

Many methods exist to calculate the MCID, but no consensus on the best approach has been reached. Both anchor- and distribution-based methods have arbitrary elements. Distribution-based methods typically use statistical characteristics of the sample, such as the standard deviation, to distinguish signal from noise. However, they do not incorporate the patient perspective, and the standard deviation can vary among patient populations, complicating determination of the clinically important size of the change. Ideally, the MCID should be linked to a clinical measure. Anchor-based methods rely on patient-perceived changes in clinical status, but they are limited by the availability of suitable anchors. The Clinical Global Impression Scale (CGI-I) is often used as an external standard in anchor-based methods because of its presumed clinical relevance. However, even this tool has limitations, since the subjective nature of patient-reported outcomes can introduce variability. In our study, we addressed these issues by using both the CGI-I as the physician-perspective anchor and the VAS as the patient-perspective anchor. With this dual approach, we aimed to provide a more comprehensive assessment of the MCID by incorporating both professional and patient perspectives.

Several studies on heterogeneous populations have attempted to determine the MCID threshold value for patients with MDD. The estimated ranges of MCIDs for the MADRS score in the present analysis (4.89–4.94 for the MADRS score, 31.15–35.10% for the MADRS reduction rate) were substantially higher than those previously reported. For instance, Duru and Fantino (3) gave MCID values for the MADRS score of 1.6–1.9. Their analysis, which included 177 placebo-treated patients from three placebo-controlled randomized trials in Europe and the US, yielded high test–retest reliability. However, only one distribution-based method (SEM) was used in that study. Kounali et al. (11) reported an MCID representing a 20% reduction in scores on the Patient Health Questionnaire-9 (PHQ-9), the Beck Depression Inventory-II, and the Generalized Anxiety Disorder Assessment-7 for patients with moderately severe depressive symptoms. In their study, a prospective cohort of 400 patients was interviewed at three primary-care sites in the UK. The authors also mentioned that if the treatment had the same effect on patients irrespective of baseline severity, those with low symptom severity were unlikely to notice a benefit. Button et al. (12) also found that the MCID could differ among patients with different types of MDD, estimating an MCID of a 17.5% reduction in scores from baseline for typical depression and a higher threshold of 32% for individuals with longer-duration depression who had not responded to antidepressants.

The higher estimated MCID values in the present study could be explained as follows. 1) The patients enrolled in this study were Japanese patients with MDD who showed an inadequate response to antidepressant monotherapy (a 17-item Hamilton Rating Scale for Depression total score ≥ 14; history of an inadequate response to 1–3 antidepressant drug treatments; and an inadequate response to 8-week, single-blind, prospective SSRI/SNRI treatment). According to Button et al. (12), this type of patient may have a higher MCID threshold than patients with typical MDD. 2) The baseline MADRS score was approximately 19, indicating that the severity of patient symptoms in the current analysis was moderate, potentially resulting in a higher MCID threshold. Kounali et al. (11) also found an increase in MCID at lower levels of severity, and it is not surprising that individuals with lower severity find it more difficult to distinguish between ‘feeling the same’ and ‘feeling better’. For these reasons, although the MCID values in the present study are higher than previous studies, the results of this study may be most relevant for patients with MDD who show an inadequate response to antidepressant therapy.

With respect to the MCID for the SDS and EQ-5D-5L-derived utility score (0.69–2.14 and 0.045–0.195, respectively), to our knowledge, this was the first report of an MCID for patients with MDD who had an inadequate response to antidepressants.

### 4.2 Differences between patient and physician perspectives

Several research groups have investigated the concordance or divergence between what physicians and patients consider the most important issues in depression treatment. A prospective, non-interventional study conducted in Belgium by Demyttenaere et al. (13) found significant differences between physicians and patients in this regard: physicians focused mainly on alleviating depressive symptoms, while patients focused mainly on quality of life and functionality. Specifically, the five most important items scored by patients were all from the World Health Organization Quality of Life Brief Version, whereas three of the five most important items according to physicians were from PHQ-9. Zimmerman et al. (14) showed that, from a patient perspective, the most important expectations for antidepressant treatment were the presence of positive mental health (optimism, vigor, self-confidence); feeling like their usual, normal self; returning to their usual level of functioning at work, home, or school; feeling in emotional control; and participating in and enjoying relationships with family and friends; the absence of depressive symptoms was ranked sixth. They developed the Remission from Depression Questionnaire (RDQ), a 41-item, self-report measure used to assess features reported by patients as relevant to determining remission from depression, including positive mood. Significant differences were found between Hamilton Depression Rating Scale-remitted patients (observer-rated) and RDQ-remitted patients (self-rated), indicating that patients with depression view remission as going beyond symptom resolution to include positive mental health and life satisfaction.

To our knowledge, this is the first formal attempt to investigate the concordance or divergence in what physicians and patients consider important in terms of disease improvement in MDD in Japan. Our results are consistent with those of Demyttenaere et al. (13) and Zimmerman et al. (14) in that important differences were revealed between what physicians and patients consider important in relief from or remission of depression. This is immediately apparent when looking at the differences in MCID values derived from patients and physicians. The MCID values for the MADRS score and MADRS reduction rate derived from the patient-perspective anchor (4.89 for the MADRS score, 34.95% for the MADRS improvement rate) and the physician-perspective anchor (4.94 for the MADRS score, 31.15% for the MADRS improvement rate) were very similar. However, the SDS and utility scores from the patient perspective were almost twice as high as those from the physician perspective, indicating that patients require bigger improvements in these areas to feel better than suggested by the physicians’ judgment. This becomes even more obvious when comparing the AUC and correlation results for the MADRS, SDS, and utility scores between the physician and patient anchors. For the patient-perspective anchor, the utility score had the highest AUC and correlation coefficients, indicating that an improvement in utility is the most accurate indicator of meaningful treatment efficacy and most responsive to patient satisfaction. For the physician-perspective anchor, the AUC for both the MADRS score and MADRS improvement rate was > 0.8, indicating good performance, whereas that of the utility and SDS scores was < 0.6, indicating that improvements in those scores failed to predict the physicians’ anchor. This suggests that physicians do not prioritize patients’ functional and utility-related improvement when assessing whether they are getting better. Shared decision-making (SDM), a patient-centered care process where patients actively participate in medical decisions, might effectively address this gap, even though it has not been widely implemented in clinical practice in Japan (15).

### 4.3 Limitations

This study had limitations that may have affected the results. First, since the data were from a phase 3 clinical trial, participation in the trial may have introduced selection biases. Because of data availability, we included only data from the long-term part of the trial, which may have caused the baseline MADRS score to be higher. It should be noted that the severity of symptoms in our sample might have influenced the MCID calculations. Specifically, the difficulty in distinguishing differences at lower severity levels might have introduced a bias in the MCID calculations. This may have limited our ability to identify the most representative MCID-calculation method, complicating assessment of whether some of the variation in MCID thresholds we found were due to statistical artifacts. Moreover, the VAS and the CGI measure slightly different underlying constructs, which may have influenced the results. Nonetheless, all four scales were assessed under the same conditions, which may have mitigated such biases. Second, the patient group was highly homogeneous, limiting the generalizability of the results. The perception of psychosocial functioning may vary among cultures and this study was only based on the Japanese trial. While, as the MCID values for the MADRS, SDS, and EQ-5D-5L scores for Japanese patients with MDD following an inadequate response to antidepressants still remain unknown, this study will be particularly valuable for clinical practice in Japan. Third, the subjective nature of MCID assessments introduces a potential for bias, as patient-reported thresholds may be influenced by individual perceptions, health literacy, or contextual factors, whereas physician-derived thresholds may reflect clinical heuristics rather than patient experiences. Finally, investigating the MCID values in patients with typical MDD is desirable, but the homogeneity of the patient group did not allow us to do so in this study.

## 5 Conclusions

The MCID values for Japanese patients with MDD who had an inadequate response to antidepressants were 4.89–4.94 for the MADRS score, 31.15–35.10% for the MADRS improvement rate, 0.69–2.14 for the SDS score, and 0.045–0.195 for EQ-5D-5L derived utility score. The MCID values for the SDS and utility scores derived from the patient-perspective anchor were higher than those derived from the physician-perspective anchor. Physicians thus focus more on depressive symptoms over daily life improvements in their assessment of global severity. In contrast, patients place greater emphasis on improvements in functionality and quality of life.

## Data Availability

The original contributions presented in the study are included in the article/supplementary material. Further inquiries can be directed to the corresponding author.
